# Angiosperm symbioses with non‐mycorrhizal fungal partners enhance N acquisition from ancient organic matter in a warming maritime Antarctic

**DOI:** 10.1111/ele.13399

**Published:** 2019-10-17

**Authors:** Paul W. Hill, Richard Broughton, Jeremy Bougoure, William Havelange, Kevin K. Newsham, Helen Grant, Daniel V. Murphy, Peta Clode, Soshila Ramayah, Karina A. Marsden, Richard S. Quilliam, Paula Roberts, Caley Brown, David J. Read, Thomas H. Deluca, Richard D. Bardgett, David W. Hopkins, Davey L. Jones

**Affiliations:** ^1^ School of Natural Sciences Bangor University Bangor LL57 2UW UK; ^2^ British Antarctic Survey Natural Environment Research Council High Cross Madingley Road Cambridge CB3 OET UK; ^3^ Institute of Aquaculture University of Stirling Stirling UK; ^4^ Faculty of Science SoilsWest UWA School of Agriculture and Environment University of Western Australia Crawley WA 6009 Australia; ^5^ Centre for Microscopy, Characterisation and Analysis University of Western Australia Crawley WA 6009 Australia; ^6^ Life Sciences Mass Spectrometry Facility Lancaster Environment Centre Lancaster LA1 4AP UK; ^7^ UWA School of Biological Sciences University of Western Australia Crawley WA 6009 Australia; ^8^ Faculty of Veterinary and Agricultural Sciences The University of Melbourne Parkville Vic. 3010 Australia; ^9^ Biological and Environmental Sciences Faculty of Natural Sciences University of Stirling Stirling FK9 4LA UK; ^10^ Animal and Plant Sciences University of Sheffield Western bank Sheffield S10 2TN UK; ^11^ WA Franke College of Forestry and Conservation University of Montana Missoula MT 59812 USA; ^12^ School of Earth and Environmental Sciences University of Manchester Manchester M13 9PL UK; ^13^ SRUC – Scotland’s Rural College West Mains Road Edinburgh EH9 3JG UK

**Keywords:** carbon cycle, climate change, dark septate endophytes, enantiomers, nitrogen cycle, polar, soil

## Abstract

In contrast to the situation in plants inhabiting most of the world’s ecosystems, mycorrhizal fungi are usually absent from roots of the only two native vascular plant species of maritime Antarctica, *Deschampsia antarctica* and *Colobanthus quitensis*. Instead, a range of ascomycete fungi, termed dark septate endophytes (DSEs), frequently colonise the roots of these plant species. We demonstrate that colonisation of Antarctic vascular plants by DSEs facilitates not only the acquisition of organic nitrogen as early protein breakdown products, but also as non‐proteinaceous d‐amino acids and their short peptides, accumulated in slowly‐decomposing organic matter, such as moss peat. Our findings suggest that, in a warming maritime Antarctic, this symbiosis has a key role in accelerating the replacement of formerly dominant moss communities by vascular plants, and in increasing the rate at which ancient carbon stores laid down as moss peat over centuries or millennia are returned to the atmosphere as CO_2_.

## Graphical abstract

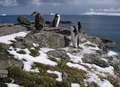

## Introduction

**Figure 1:**
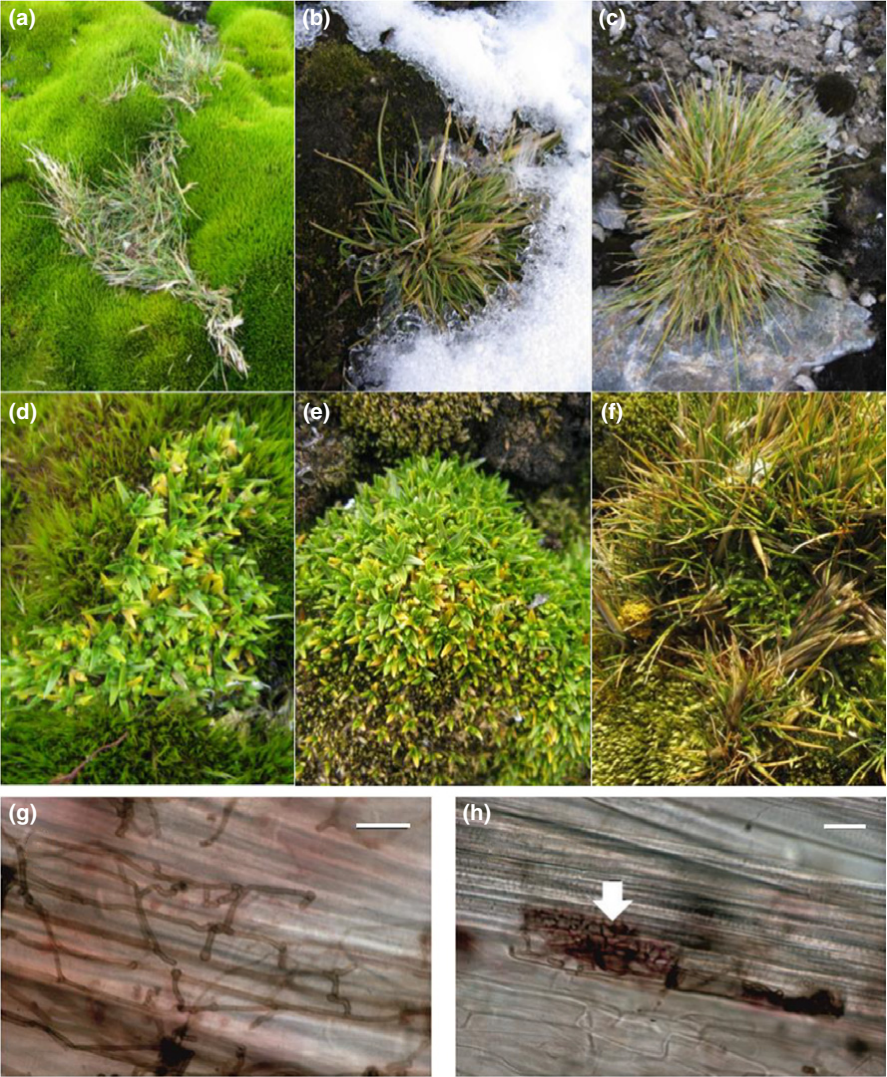
Antarctic vascular plants exploiting areas previously colonised by mosses on Signy Island and DSEs in roots of *Deschampsia antarctica*. (a) *D. antarctica* growing in a bank of *Chorisodontium aciphyllum*. (b) *D. antarctica* growing through mixed *Sanionia uncinata* and *Polytrichum juniperinum*. (c) *D. antarctica* growing among *Andreaea* sp. (d) *Colobanthus quitensis* growing through *C. aciphyllum*. (e). *C. quitensis* growing through *S. uncinata*. f. *D. antarctica* and *C. quitensis* growing with *S. uncinata*. (g) DSE hyphae in *D. antarctica* root. (h) DSE microsclerotium (arrowed) in *D. antarctica* root (scale bars on panels g and h are 20 µm).

Fungal root symbionts have been crucial to the success of plants in terrestrial ecosystems, with a relationship dating back to the colonisation of land (Strullu‐Derrien *et al.* 2018). Mutualistic relationships with mycorrhizal fungi remain key to the acquisition of limiting nutrients, such as nitrogen (N) and phosphorus (P), in the majority of terrestrial plants (Smith & Read 2008). However, in marked contrast to their presence in most ecosystems, mycorrhizas are typically absent from the roots of vascular plants in maritime Antarctica (Upson *et al.* 2008; Newsham *et al.* 2009). In this region, the roots of the two native angiosperms, *Deschampsia antarctica* Desv. (a grass) and *Colobanthus quitensis* (Kunth) Bartl. (a cushion‐forming plant, Fig. 1) are instead colonised by a range of ascomycete fungi, collectively termed dark septate endophytes (DSEs) (Fig. 1; Upson *et al.* 2008; Newsham *et al.* 2009), which may have a role in the acquisition of organic N from soils (Upson *et al.* 2009; Newsham 2011).

In areas of the maritime Antarctic not under permanent ice, moss cover can be extensive (Fig. S1) and dominates primary productivity. It is estimated to account for 45 km^2^ of Antarctic Peninsula land area and is particularly prevalent on islands such as the South Orkney and South Shetland Islands (Fretwell *et al.* 2011; Royles & Griffiths 2015). Due to the constraints imposed on decomposition by low temperatures, moss growth leads to the accumulation of large amounts of soil organic matter, including substantial stores of protein (Royles & Griffiths 2015). Vascular plants, and particularly pioneer individuals and populations, are commonly found among mosses, exploiting stored proteinaceous N to facilitate establishment (Fig. 1, Hill *et al.* 2011a).

In a survey of roots of *D. antarctica* and *C. quitensis* on Signy Island (60° 43’S, 45° 36’W) in the South Orkney Islands, maritime Antarctica, we found the most consistent and extensive occurrence of DSE hyphae and characteristic microsclerotia (Fig. 1) was in the roots of plants growing among banks formed by the moss *Chorisodontium aciphyllum* (Hook. f. & Wilson) Broth. (Fig. 1). Banks formed by this moss frequently exceed 1 m in depth and may be up to 3 m deep, storing organic matter that has remained undecomposed over millennia (Royles *et al.* 2012; Royles & Griffiths 2015; Fig. S2). This organic matter has become increasingly bioavailable as mean air temperatures have risen in the maritime Antarctic, leading to progressive thawing of the moss banks (Royles *et al.* 2012; Abrams *et al.* 2013; Royles & Griffiths 2015; Amesbury *et al.* 2017).

In most cases (e.g. among the moss *Sanionia uncinata* (Hedw.) Loeske, Fig. 1 and Fig. S1), *D. antarctica* appears to root no deeper than *c*. 10 cm, with its roots usually extending to a depth of 5 cm or less (Fig. S3), corresponding to the depth of accumulated organic matter. However, in *C. aciphyllum* banks, the grass was observed rooting down to > 25 cm, where organic matter may have been stored for > 500 years (Royles *et al.,* 2012). We hypothesised that the penetration of roots colonised by DSEs deep into moss banks allows *D. antarctica* to exploit ancient nutrients that up until recent decades were unavailable because the moss banks have been frozen.

Due to slow N mineralisation, it is likely that early breakdown products of accumulated proteins (l‐amino acids and their short peptides) make a substantial contribution to plant N nutrition in polar soils (Chapin *et al.* 1993; Hill *et al.* 2011a). However, peptides containing d‐glutamic acid and especially d‐alanine are common constituents of bacterial peptidoglycan and various d‐amino acids occur in bacteria, archaea, fungi, plants and animals (Yoshimura & Esaki 2003; Friedman 2010; Vranova *et al.* 2012). d‐amino acids are also known to accumulate from proteinaceous l‐amino acids during long periods of storage, due to abiotic racemisation, which may take place at a rate of about 0.3% of l‐amino acids per decade (Wichern *et al.* 2004). Consequently, d‐amino acids accumulate in soils where decomposition is slow e.g. in deserts or in peat, such as that formed by moss banks (Kunnas & Jauhiainen 1993; Wichern *et al.* 2004).

It is clear from previous investigations that both plants and soil microbes are able to take up and metabolise some d‐amino acids such as d‐alanine (Hill *et al.* 2011b,c; Hill *et al.* 2012; Vranova *et al.* 2012). However, in contrast to short l‐peptides, which appear to be widely metabolised, until now, evidence suggested that short d‐peptides could be metabolised by soil microbes but not by plants (Hill *et al.* 2011b,c; Hill *et al.* 2012; Vranova *et al.* 2012). Whether the ability to metabolise d‐peptides is present in plants inhabiting soils where d‐enantiomers are a more available source of N is unknown. We measured uptake of a range of N forms under field conditions in the Antarctic and found that both native vascular plants could acquire N from d‐alanine and its dipeptide – as well as from longer peptides of the l‐enantiomer than previously recognised. Further, we found that colonisation with DSEs facilitated plant acquisition of N from both l‐ and d‐enantiomers of alanine and their peptides.

## Materials and methods

### Assessment of fungal endophyte colonisation

Roots of *D. antarctica* and *C. quitensis* were collected from locations around Signy Island (Gourlay Peninsula; Polynesia Point; Factory Cove; Berntsen Point; Lower slopes of Factory Bluffs; Starfish Cove; North Point; Moss Braes; Deschampsia Point; Foca Cove; Fig. S4). Roots were washed in water and examined for the presence of DSE hyphae and microsclerotia by light microscopy after staining (Newsham & Bridge 2010). The same analyses confirmed the absence of arbuscular mycorrhizal structures from roots (Upson *et al.* 2008).

### Soil solution collection

Rhizon soil solution samplers (5 cm long; Rhizosphere Research Products, Wageningen, Netherlands) were inserted into soil under mosses (mostly *S. uncinata* and *C*. *aciphyllum*) or vascular plants (*D. antarctica* with some *C. quitensis*). Soil solution was sampled over a depth of *c*. 2–6 cm at approximately fortnightly intervals for about 12 weeks during austral summer. Large soluble proteins and peptides were then removed by passing solutions through a 1 kDa ultrafiltration membrane (Millipore, Billerica, MA, USA).

### Analysis of amino acid enantiomers

Filtered soil solution samples taken over the season from each site were pooled, divided in two and concentrated by freeze drying. One portion was hydrolysed for 16 h in 6 M HCl under N_2_ and freeze‐dried again. The dry soil solution residues were re‐suspended in 500 μL of 0.01 M HCl with 1.875 pmol μL^−1^ of l‐homoarginine as the internal standard. Amino acid enantiomers were quantified by HPLC (Broughton *et al.* 2015).

### Substrate uptake in intact plant‐soil system

Monoliths (*c*. 20 × 20 cm) of *D. antarctica* or *C. quitensis* growing in native soil were collected from the Moss Braes region of Signy Island and stored outside for about 24 h prior to experiments. About 1–2 h prior to experiments, 15 mm diameter, 40 mm deep plugs were taken from the monoliths. Solutions (2.5 mL) of 98 at% ^15^N (inorganic) or dual ^15^N, ^13^C (organic) 1 mM l‐alanine, d‐alanine, l‐dialanine, d‐dialanine, l‐trialanine, l‐tetraalanine, l‐pentaalanine, NH_4_Cl or KNO_3_ (l‐enantiomers, and inorganic from CK‐Gas Products, Hook, UK; d‐enantiomers from Sigma‐Aldrich, Gillingham, UK) were injected into plugs (*n* = 4 and *n* = 3 for *D. antarctica* or *C. quitensis*, respectively). After 1 h in daylight at *c*. 2 °C, shoot material was removed, dried (80 °C) and ground before analysis in a Eurovector Isoprime IRMS (Eurovector SpA, Milan, Italy).

### Sterile culture of *D. antarctica* and inoculation of roots with DSEs

Sterile individuals of *D. antarctica* (we were not able to generate a sterile culture of *C. quitensis*) were prepared according to a protocol modified from Cuba *et al. *(2005). Plants were removed from soil and washed in tap water. Roots and shoots were trimmed and the remaining tissue was shaken in NaHClO_3_ (*c*. 14% free Cl) with 1 drop of Tween 20 for 25 min, followed by 80% ethanol for 5 min. After thorough washing in sterile tap water, the remaining leaf and root was trimmed from crown tissue, which was then placed on the surface of sterile agar containing 2.1 g l^−1^ Murashige & Skoog basal medium, 1 mmol L^−1^ glucose and 47 µmol L^−1^ NaSiO_3_ in Phytatrays (Sigma‐Aldrich, Gillingham, UK). Amphotericin B solution (5 mL of 2.5 mg L^−1^) was then added to the surface of agar around the crown tissue. Plants were grown at 10 °C with a 16 h photoperiod at *c*. 500 µmol photons m^−2^ s^−1^. Tillers were separated periodically and replanted in agar as above (except for amphotericin B, which was not used after the first culture). Any Phytatrays showing signs of microbial contamination were discarded. Examination of roots of sterilised plants by light microscopy and TEM did not reveal the presence of any microbes.

Sterile plants for use in experiments were transplanted into Phytatrays containing sterile perlite with *c*. 100 mL of 2.1 g L^−1^ Murashige & Skoog basal medium, 1 mmol L^−1^ glucose and 47 µmol L^−1^ NaSiO_3_ with and without inoculation with a DSE (*Tapesia* sp.; Helotiales; GenBank accession #FN178471), which was isolated from roots of *D. antarctica* growing on Coronation Island, around 7 km from where experimental plants and soils were collected. At least three weeks was allowed for the DSE to colonise roots before plants were used in experiments. Plants were then removed from the inoculated perlite and grown in uninoculated perlite, as used for the controls.

### Substrate uptake from sterile solution

Sterile or DSE‐inoculated *D. antarctica* plants were removed from perlite and roots gently washed in sterile 0.1 mM KCl, followed by deionised water. Roots of intact plants (*n* = 4) were then placed in sterile vials containing 2 mL of 100 µM, 98 at% ^15^N (inorganic) or dual ^15^N, ^13^C (organic) l‐alanine, d‐alanine, l‐dialanine, d‐dialanine, l‐trialanine, d‐trialanine, l‐tetraalanine, l‐pentaalanine, NH_4_Cl or KNO_3_. After 1 h, plants were removed from solutions, washed in deionised water followed by 100 mM CaCl_2_. Roots and shoots were separated and analysed by IRMS, as above.

### Plant metabolism of substrates

To determine whether substrates could be metabolised, sterile or DSE‐inoculated roots of intact *D. antarctica* plants (*n* = 3) were submerged in 2 mL of 10 µM, *c*. 7.5 kBq mL^1^ 1‐^14^C l‐alanine, d‐alanine, l‐dialanine, d‐dialanine, l‐trialanine, d‐trialanine, l‐tetraalanine or l‐pentaalanine (American Radiolabeled Chemicals, St Louis, MO, USA). Vials and plants were sealed in 50 mL clear polypropylene containers. Air was drawn through containers at 300 mL min^−1^ and bubbled through 15 mL Oxysolve C‐400 Scintillant (Zinsser Analytic, Frankfurt, Germany) to capture respired ^14^CO_2_. Carbon dioxide traps were changed after 10, 20, 40, 60 and 80 min and captured ^14^CO_2_ measured by scintillation counting in a Wallac 1404 scintillation counter (Perkin‐Elmer Life Sciences, Waltham, MA, USA).

After 80 min, plants were removed from solutions, washed as above and dried. Dry roots and shoots were combusted in a Harvey OX400 Biological Oxidiser (Harvey Instruments Corp., Hillsdale, NJ, USA). Liberated ^14^CO_2_ was captured in Oxysolve C‐400 and ^14^C activity measured by liquid scintillation counting as above.

### Uptake kinetics

Sterile or DSE‐inoculated roots of intact *D. antarctica* plants (*n* = 3) were submerged in labelled (^14^C or ^15^N for organic and inorganic substrates, respectively) substrate solutions as above. In this case, exposure to solutions was for 15 min and substrate concentrations were 1, 5, 10, 50, 100, 250, 500, 750 µM and 1, 2.5, 5, 7.5 and 10 mM. Plants were analysed for ^14^C or ^15^N as above. Respired ^14^CO_2_ was captured in Oxysolve C‐400 and measured as above. Michaelis–Menten constants were calculated from hyperbolic fits to uptake data (Sigmaplot v13, Systat, Hounslow, UK).

### NanoSIMS analysis

Sterile or DSE colonised *D. antarctica* (*n* = 3) roots were submerged in 3 mM solution of either ^13^C^15^N d‐trialanine or ^13^C^15^N ‐l‐trialanine. Plants were incubated for 5 mins, removed from isotope enriched solution, washed quickly in MQ water and then high pressure frozen (HPF; 1 mm segments) in hexadecene cryoprotectant (EM PACT2, Leica Microsystems, Wetzlar, Germany). HPF samples were cryosubstituted (EM AFS2, Leica Microsystems, Wetzlar, Germany) using the method described in Bougoure *et al. *(2014). Briefly, samples were immersed in prechilled (−130 °C) acrolein:diethyl ether over molecular sieve and brought to room temperature over 3 weeks before being infiltrated and embedded in epoxy resin. Sections 250 nm thick were cut dry (i.e. not floated onto water for collection), mounted on Si wafers, and Au coated (10 nm) for nanoSIMS analysis. Regions of interest were identified and imaged at 120 kV in a transmission electron microscope (TEM; JEOL 2100) fitted with a digital camera (Gatan, ORIUS1000; Gatan Inc., Pleasanton, CA, USA). Sections were also collected on glass slides, stained with toluidine blue and examined by optical microscopy to guide locations of nanoSIMS analyses.

*In situ* isotopic mapping was done using a NanoSIMS 50 (Cameca, Gennevilliers, France), with a 16 keV Cs^+^ primary ion beam. Analyses were performed in multi‐collection mode simultaneously detecting negative secondary ions ^12^C_2_, ^12^C^13^C, ^12^C^14^N, and ^12^C^15^N. The mass spectrometer was tuned to high mass resolution of *c*. 10 000 (CAMECA definition) to separate ^12^C^15^N from ^13^C^14^N using an entrance slit of 30 µm, an aperture slit of 200 µm, and a 10% reduction in the signal at the energy slit. For secondary ion imaging, the primary current was set to *c*. 2 pA using a 350‐µm primary aperture, giving a spot size of *c*. 100 nm. Analyses were done in chain mode so individual 30 × 30 µm analyses (256 pixel resolution) could be montaged to generate a dataset across entire root sections. All areas were implanted to the same ion dose (6 × 10^16^ ions cm^−2^) prior to each acquisition.

Images were processed using the OpenMIMS data analysis software (National Resource for Imaging Mass Spectrometry http://nrims.harvard.edu) for the freeware package ImageJ (National Institutes of Health, Bethesda, MD, USA). Images were corrected for detector dead time (44 ns) on individual pixels and montages were produced using NRRD mosaics script (http://nrims.harvard.edu).

### Statistical analyses

Data were analysed by *t*‐test, one‐way anova with Tukey HSD post hoc test or repeated measures anova (SPSS v22; IBM, New York, NY, USA) after testing for normality and homogeneity of variance with Shapiro–Wilk and Levene’s tests, respectively. Data not conforming were transformed prior to analysis. Where a suitable transformation could not be identified, Games–Howell test was used. Statistical differences were accepted at *P* ≤ 0.05 unless otherwise stated.

## Results

### Amino acid concentrations in soil solution

**Figure 2:**
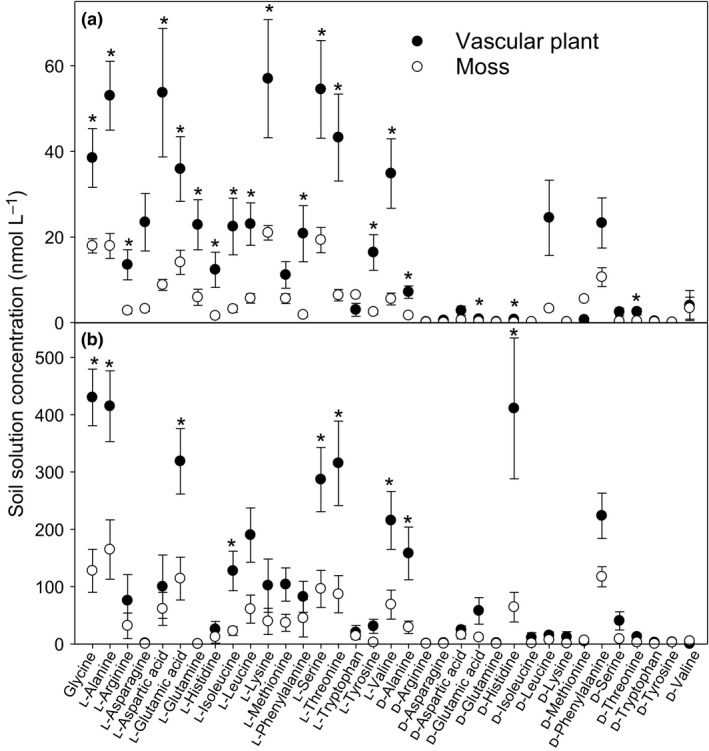
Concentrations of d‐ and l‐enantiomers of amino acids in soil solutions at Signy Island under mosses alone or where vascular plants are present. (a) free amino acids. (b) amino acids bound in soluble peptides. Values are means ± SEM; *n* = 23 and *n* = 16 for free and bound amino acids, respectively, under vascular plants; *n* = 26 and *n* = 21 for free and bound amino acids, respectively, under mosses only. Asterisks indicate differences between soil where vascular plants are present or where mosses are present alone (*P* ≤ 0.05).

The presence of vascular plants was associated with increases (*P ≤ *0.05) in soil solution concentrations of 16 out of 18 measured free amino acids (l‐enantiomers and glycine) by as much as 10‐fold compared to sites where mosses grew alone (Fig. 2). The concentrations of non‐protein d‐amino acids were more variable, but there was more than three times as much free d‐alanine, d‐glutamate, d‐histidine and d‐threonine (*P ≤ *0.05) in soil with vascular plants compared to moss‐only soil (the concentrations of three other d‐amino acids were greater with statistical significance at *P* < 0.1). Soluble, peptide‐bound amino acids tended to be present in soil solution at concentrations approximately ten‐times greater than free amino acids (statistically different at *P ≤ *0.05 for 20 and 21 amino acid enantiomers under vascular plants and mosses, respectively). The concentrations of almost half of the bound l‐amino acids and d‐alanine and d‐histidine were greater (*P ≤ *0.05) when vascular plants were present, relative to mosses alone.

### Uptake of amino acids and peptides under field conditions

**Figure 3:**
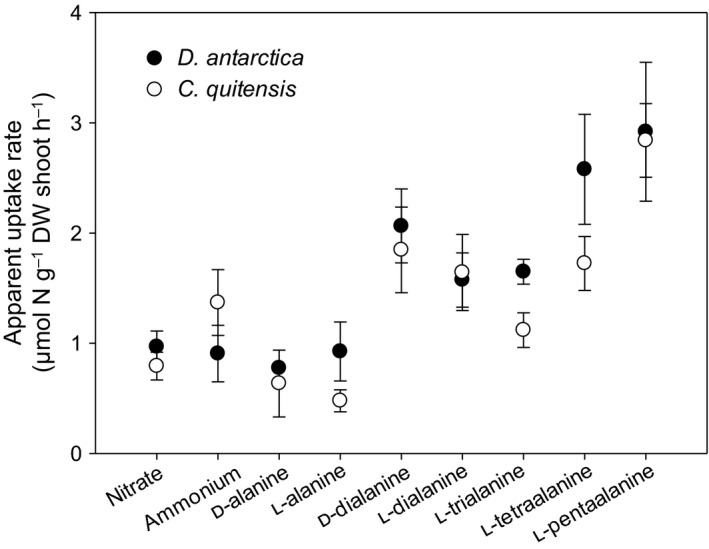
Rates of uptake of inorganic N and d‐and l‐enantiomers of alanine and short peptides thereof into shoots of *D. antarctica* and *C. quitensis* following injection of ^15^N‐ and ^13^C‐ labelled substrates into soil. Values are mean ± SEM; *n* = 3 or 4

Tests of uptake of a range of N forms under field conditions in the Antarctic showed that both native vascular plant species could acquire ^15^N from d‐alanine and its dipeptide – as well as from peptides of the l‐enantiomer up to five amino acids in length (Fig. 3). Rates of uptake appeared similar between the two species. Recovery of amino acid and peptide ^13^C suggested some intact uptake of molecules, although lack of data for root material and losses of ^13^C in respiration prevented quantification (Fig. S5). Although DSEs were present in the roots of plants used in these experiments, whether the fungal endophytes influenced nutrient acquisition could not be established.

### Uptake, partitioning and metabolism of amino acids and peptides by plants with sterile roots or colonised with DSEs

**Figure 4:**
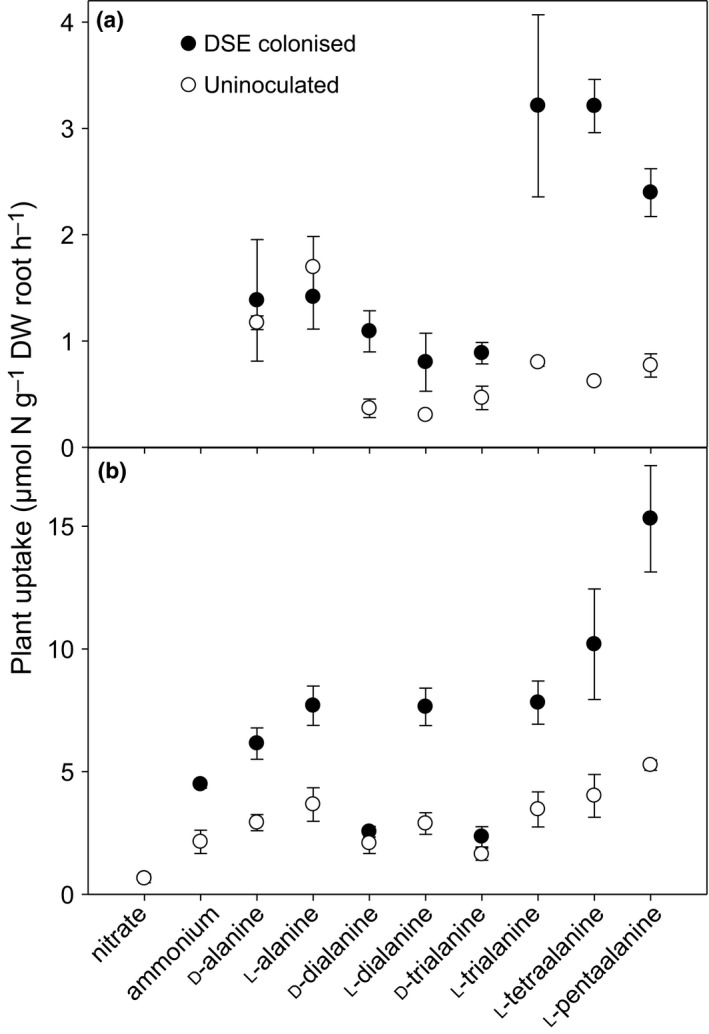
Rates of uptake by *D. antarctica* of N supplied in different forms. N uptake calculated from recovery of ^14^C (a) and ^15^N (b). Data are mean ± SEM; *n* = 3 and *n* = 4 for ^14^C and ^15^N, respectively. Calculation of N flux from ^14^C assumes that C and N entered the plant (or plant and fungus) together without extracellular separation of C and N. ^13^C data did not account for respiratory losses and are not shown.

Although there were minor differences between isotopic tracers, with the exception of nitrate, DSE colonisation increased the uptake of all forms of N supplied to roots, with strong positive effects of the endophyte on the uptake of l‐tri‐, l‐tetra‐ and l‐pentaalanine (*P* < 0.05; Fig. 4). Nitrate was also the only tested form of N where Michaelis–Menten constants for N uptake showed no indication of an effect of DSE colonisation (Table S1).

Surprisingly, the DSE appeared to promote N translocation such that colonised plants had a lower ratio of root ^15^N to shoot ^15^N than uninoculated control plants (*P* < 0.001; Fig. S6). Further, in contrast to limited data for other plants, loss of ^14^CO_2_ in respiration demonstrated that *D. antarctica* could metabolise all forms of organic N supplied, including d‐peptides (Fig. S7; Hill *et al.* 2011c). However, actual rates of C loss in respiration are probably somewhat overestimated due to the ^14^C label being located only on the carboxyl group (Dippold & Kuzyakov 2013; Hill & Jones 2019).

**Figure 5:**
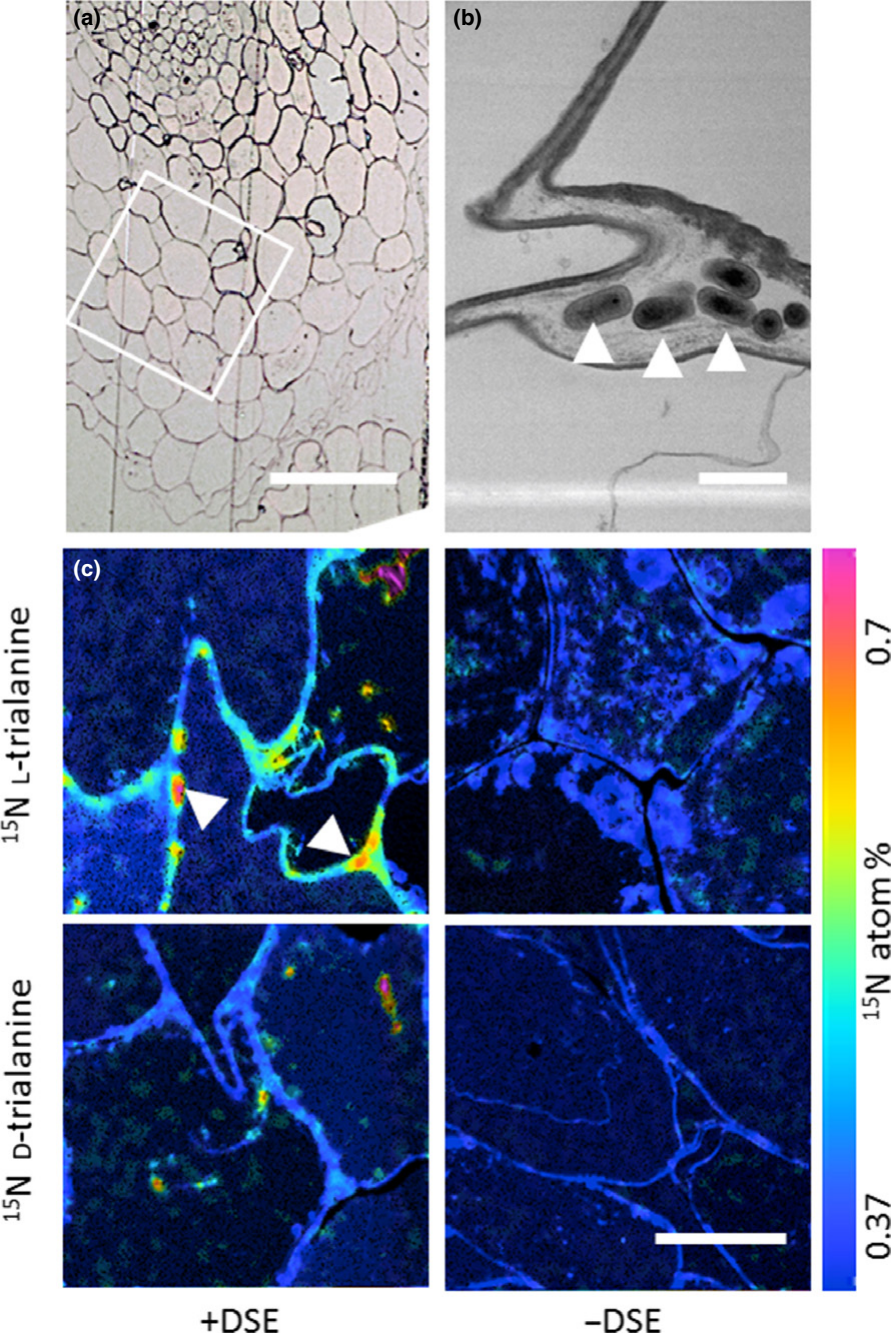
^15^N distribution within *D. antarctica* roots with and without DSE colonisation after 5 min incubation in either d or l enantiomers of ^15^N trialanine. (a) Optical image of partial DSE‐inoculated root cross‐section showing typical cell zonation, specifically the cortex (white inset square) from where nanoSIMS images (c) are taken; scale bar 100 µm. (b) TEM of intercellular space between root cortical cells of a DSE‐inoculated root showing the presence of abundant hyphae (white arrows); scale bar 2 µm. (c) The ^15^N atom percent images (nanoSIMS) of typical cortical cells in roots with or without DSE and incubated with either d or l forms of ^15^N trialanine. Highest ^15^N enrichment was observed in DSE colonised roots supplied with l‐trialanine. White arrows indicate intercellular hyphae where they can be clearly identified. Cells of DSE colonised roots supplied with d‐trialanine also showed enrichment, but hyphae could not be located with confidence. Roots without DSE showed negligible ^15^N enrichment; scale bar 10 µm.

Nanoscale Secondary Ion Mass Spectrometry (nanoSIMS) showed transfer of l‐peptide ^15^N into the intercellular space between the root cortical cells of *D. antarctica* by DSE hyphae (Fig. 5; Fig. S8). Additionally, individual root cells of plants supplied with d‐ or l‐trialanine were more enriched with ^15^N when colonised with the DSE than in sterile controls, strongly suggesting that enhanced isotope recovery in bulk root analyses was not merely separate uptake by roots and fungus.

## Discussion

It appears that the presence of vascular plants in the organic soils of the maritime Antarctic gives rise to a marked increase in availability of both l‐ and d‐enantiomers of amino acids as N sources. This suggests a stimulation of the rate of breakdown of stored moss peat in the presence of roots, probably resulting from rhizosphere priming (Gavazov *et al.* 2018). Of free (and peptide‐bound) d‐amino acids, d‐alanine was among the most available, maintaining concentrations around 10% of those of l‐alanine, despite microbial consumption at rates similar to those of l‐amino acids, indicating a significant production flux in these soils (Hill *et al.* 2011b). Whether this d‐alanine originates primarily from peptidoglycan, abiotic racemisation of l‐alanine in stored proteins, or another process is currently unknown. Similarly, although we can attribute occurrence of other d‐amino acids to racemisation, it is not clear whether this is the only or even the principal source (Vranova *et al.* 2012). However, irrespective of the exact origin, the actual increase in availability of amino acid‐N driven by vascular plants is likely to be greater than the increase in measured soil solution concentrations, due to a probable higher consumption flux from both microbes and plant roots in soils under vascular plants than under mosses (Hill *et al.* 2011a,b).

DSEs are widespread in plant roots in a range of ecosystems (Jumpponen 2001; Newsham *et al.* 2011), but there has been limited identification of their roles in plant nutrient acquisition to date, with some appearing to have negative effects on plant hosts (Jumpponen 2001; Upson *et al.* 2009; Newsham 2011; Vergara *et al.* 2018). Consequently, it remains unknown whether symbioses with DSEs are widespread facilitators of nutrient acquisition. It is clear from the findings here that the colonisation of roots by DSEs has a marked effect on the ability of Antarctic angiosperms to exploit amino acid N. The nanoSIMS images demonstrate direct hyphal transfer of peptide N to the root, and the surprising effect of DSE colonisation on translocation of N suggests an additional physiological effect on the host plant (direct hyphal transfer to shoots is unlikely due to confinement of this group of fungi to roots; Rodriguez *et al.* 2009). Colonisation appears to aid acquisition of some forms of N, such as peptides of d‐amino acids and an l‐pentapeptide, which have not previously been recognised as viable sources of N for plants. This may be due to the probable higher availability of both l‐ and d‐enantiomers in ecosystems where large quantities of proteinaceous material accumulate and turn over slowly (Chapin *et al.* 1993; Kunnas & Jauhiainen 1993; Wichern *et al.* 2004). The occurrence of close relatives of the DSE used here in the Arctic may support this view (Genbank accessions MF920427 and KF617231; Taylor *et al.* 2014; Krishnan *et al.* 2018). However, as both d‐ and l‐peptides do exist in other ecosystems and investigation into plant use of d‐peptide N has been limited, it may be that the use of these N forms by both plants and DSEs is more widespread than is currently recognised (Friedman 2010; Hill *et al.* 2011c; Vranova *et al.* 2012). Some mosses are also colonised by endophytic fungi, but there is no evidence for a role of these endophytes in nutrient acquisition (Davey & Currah 2006).

As greenhouse gas emissions to the atmosphere continue, near‐surface air temperatures in the maritime Antarctic are projected to warm by 2–4 °C by 2100 (Bracegirdle *et al.* 2008). Our measurements suggest that vascular plants could increase rates of organic matter breakdown under Antarctic mosses by up to an order of magnitude. Rising air temperatures are known to synergistically increase rhizosphere priming, with increases in temperature sensitivity of, perhaps, 25–50% in the presence of living roots (Boone *et al.* 1998; Zhu & Cheng 2011; Hill *et al.* 2015). Hence, it appears that priming of ancient organic matter stored in moss banks arising from plant growth and warming may interact to further increase nutrient availability, enhancing the proliferation of angiosperms and returning more C to the atmosphere in a complex positive feedback (Convey & Smith 2006; Day *et al.* 2008; Cannone *et al.* 2016; Gavazov *et al.* 2018; Newsham *et al.* 2018). Thus, it seems probable that the stocks of moss‐derived organic matter accumulated over millennia will disappear at increasingly rapid rates as temperatures rise and the ecology of the maritime Antarctic changes.

## Author contributions

PH, DJ, KKN, RDB, DH, PR, TD and RQ conceived the investigation; PH carried out fieldwork; RB carried out amino acid analysis; JB, DM and PC carried out nanoSIMS work; PH, WH, CB, SR and KM carried out laboratory experiments and analysis; HG carried out IRMS analysis; PH wrote the manuscript first draft; all authors contributed to the final version.

## Data Availability

Data will be archived at Figshare Repository: https://doi.org/10.6084/m9.figshare.9791693.
