# Supplementary material for: Angiosperm symbioses with non‐mycorrhizal fungal partners enhance N acquisition from ancient organic matter in a warming maritime Antarctic
Source: Ecol Lett. 2019 Oct 17;22(12):2111–9. doi: 10.1111/ele.13399 (PMC6899649; doi:10.1111/ele.13399)
Supplement: Supplementary file 1 [file ELE-22-2111-s001.pdf]

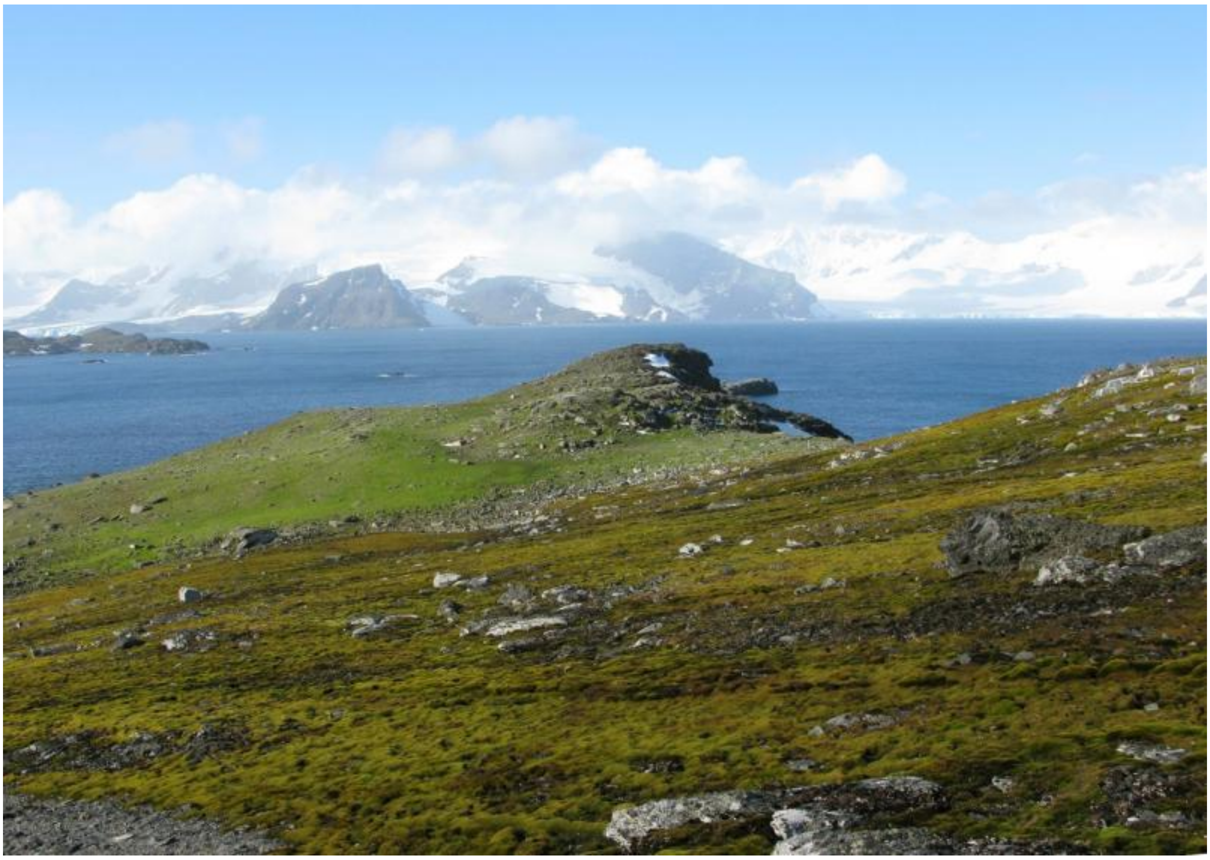

Figure S1 Moss carpet dominated by *Sanionia uncinata* on Signy Island.

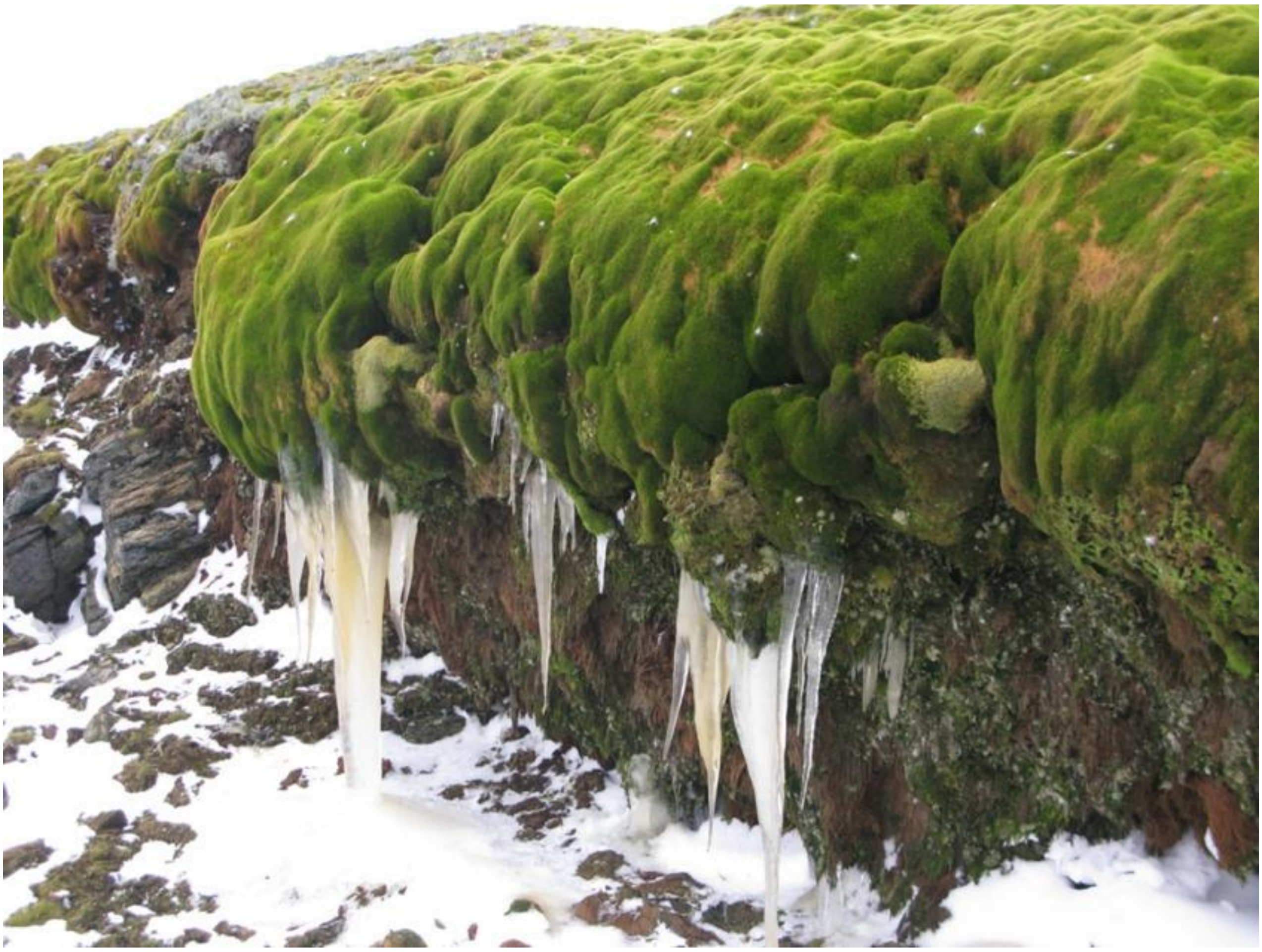

Figure S2 Moss banks on Signy Island, showing living *Chorisodontium aciphyllum* with accumulated moss peat underneath.

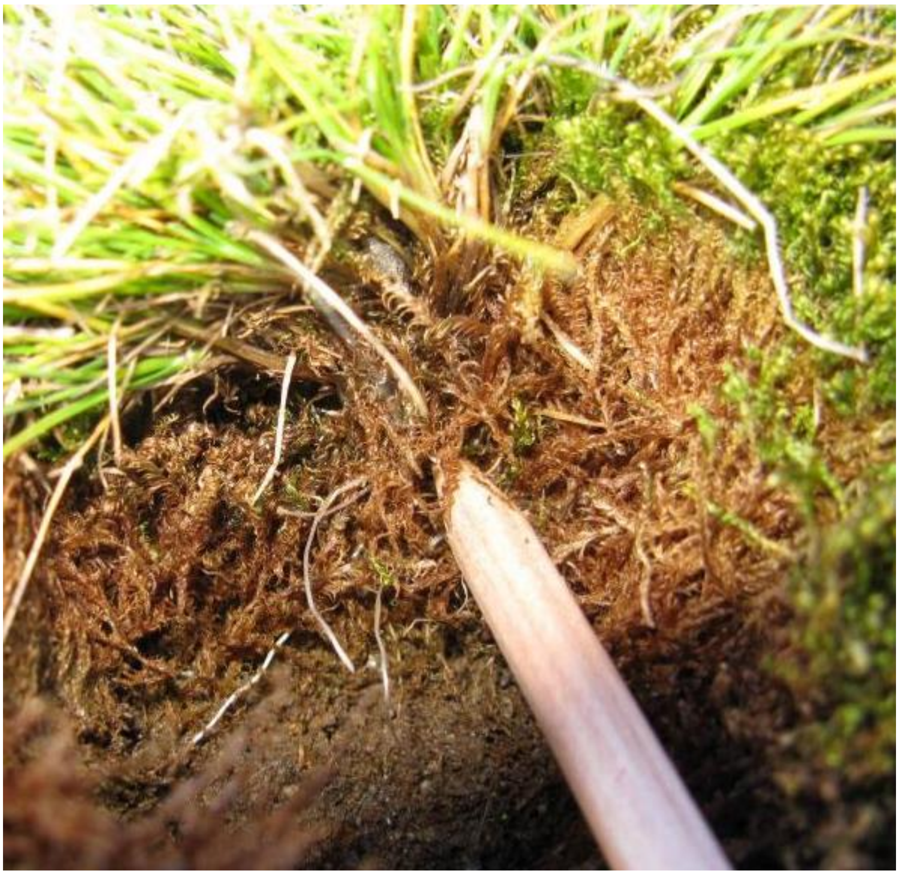

Figure S3 *Deschampsia antarctica* with shallow roots penetrating into accumulated organic matter under *Sanionia uncinata*. Pencil gives scale (*c*. 6 mm diameter).

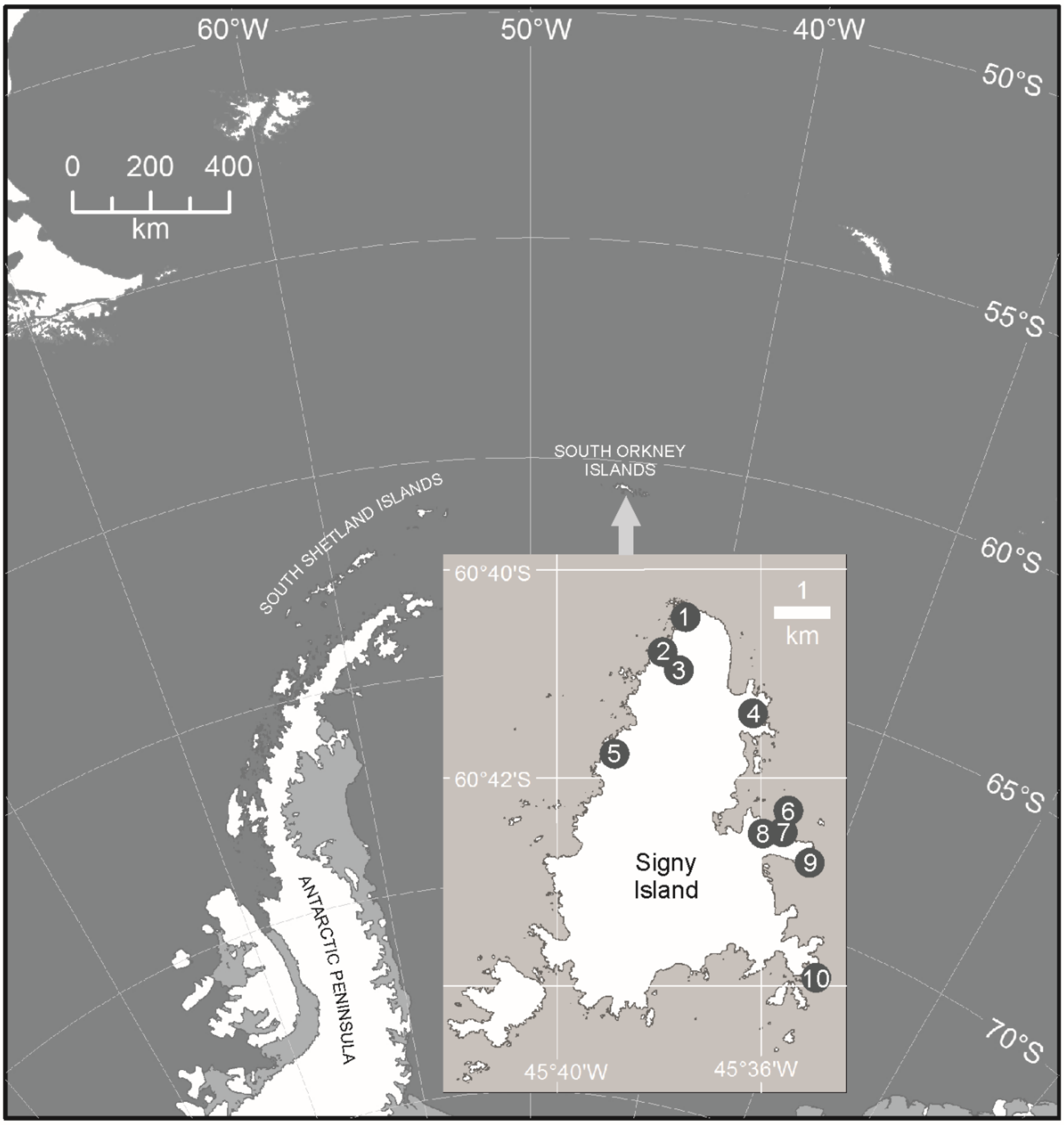

Figure S4 Location of Signy Island (inset) in maritime Antarctica. Plants were sampled from (1) North Point, (2) Deschampsia Point, (3) Moss Braes, (4) Starfish Cove, (5) Foca Cove, (6) Berntsen Point, (7) Factory Cove, (8) Factory Bluffs, (9) Polynesia Point and (10) Gourlay Point.

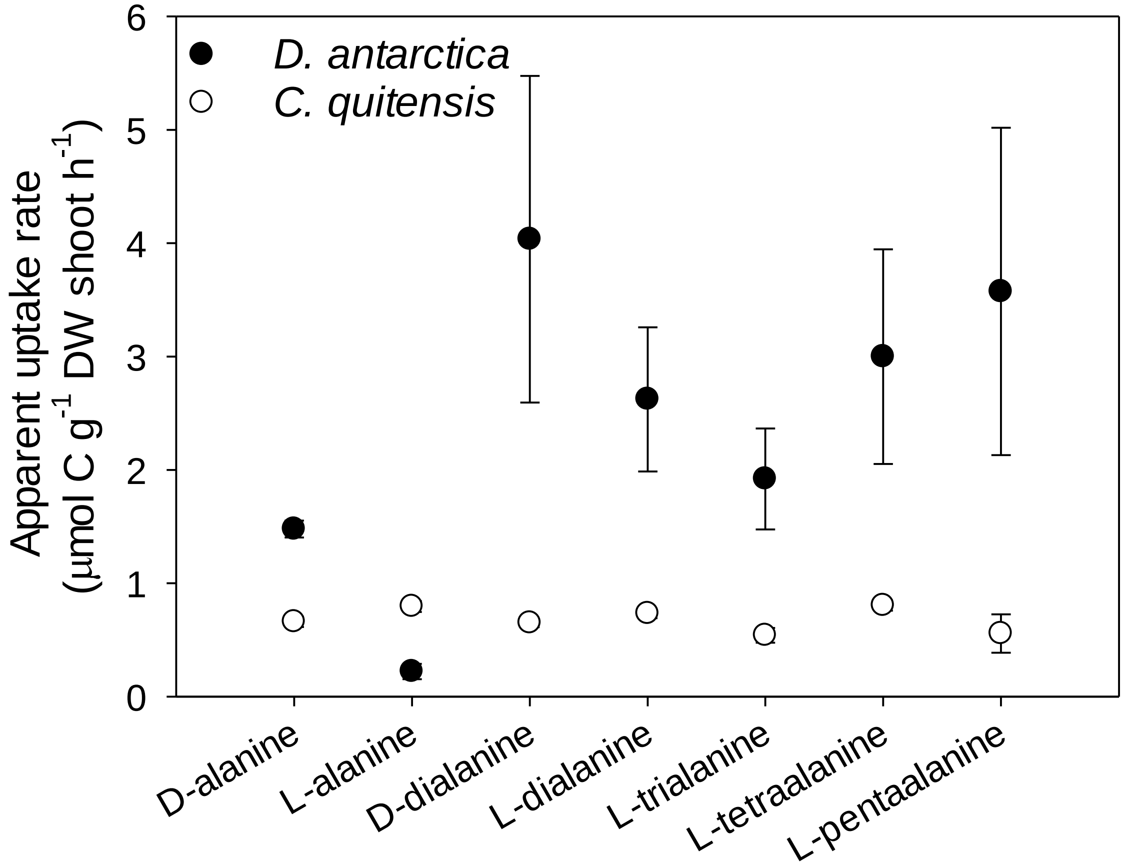

Figure S5 Apparent rates of uptake of C from D-and L-enantiomers of alanine and short peptides thereof into shoots of *D. antarctica* and *C. quitensis* following injection of ^{15}N- and ^{13}C- labelled substrates into soil. Values are mean ± SEM; *n*=3 or 4. Caution should be exercised in interpretation as differences in partitioning and losses of ^{13}C in respiration are not accounted for.

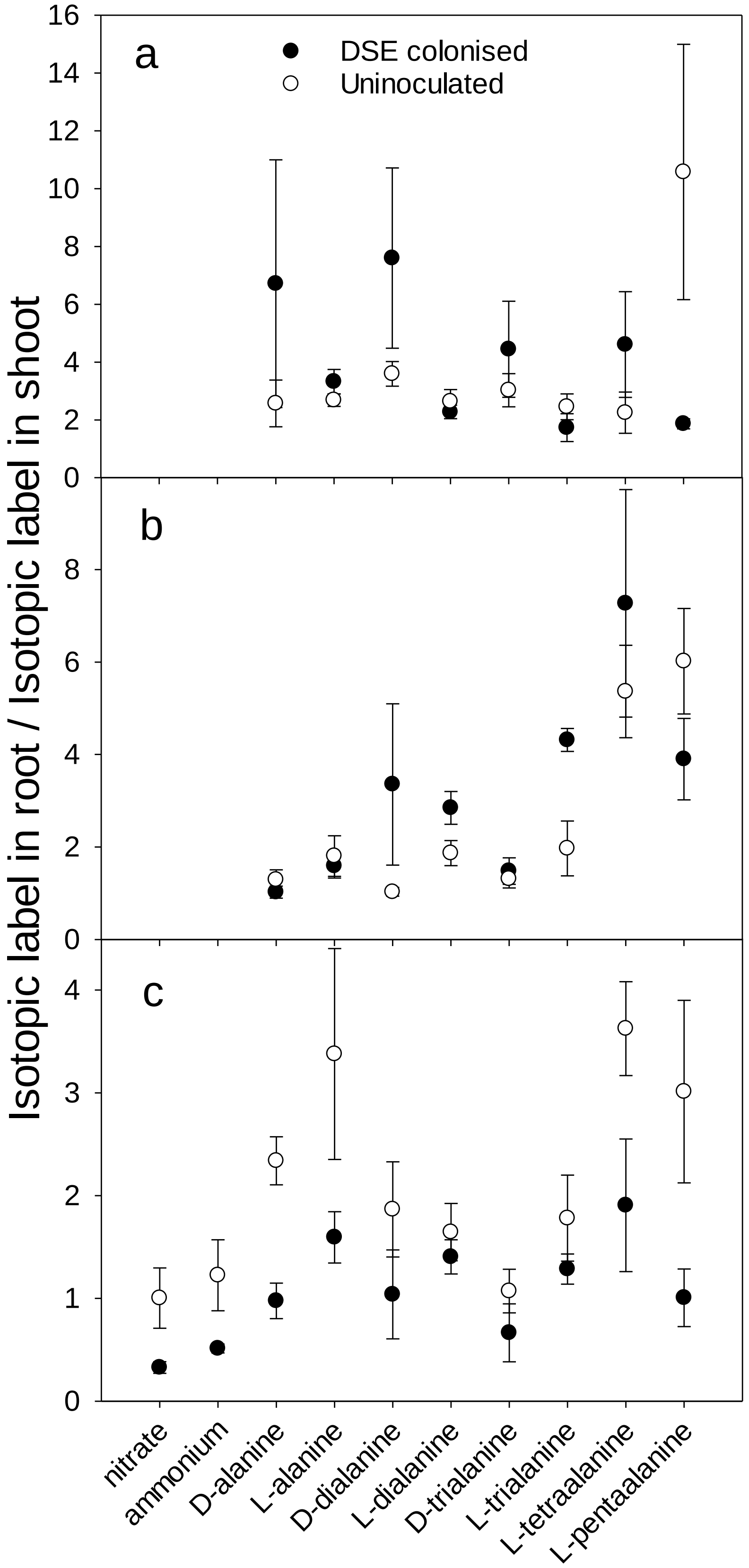

Figure S6 Ratio of recovery of isotopic labels in roots to recovery in shoots of *D. antarctica*. a. ^{14}C. b. ^{13}C. c. ^{15}N. Data are mean ± SEM; *n*=3 for ^{14}C; *n*=4 for ^{13}C and ^{15}N.

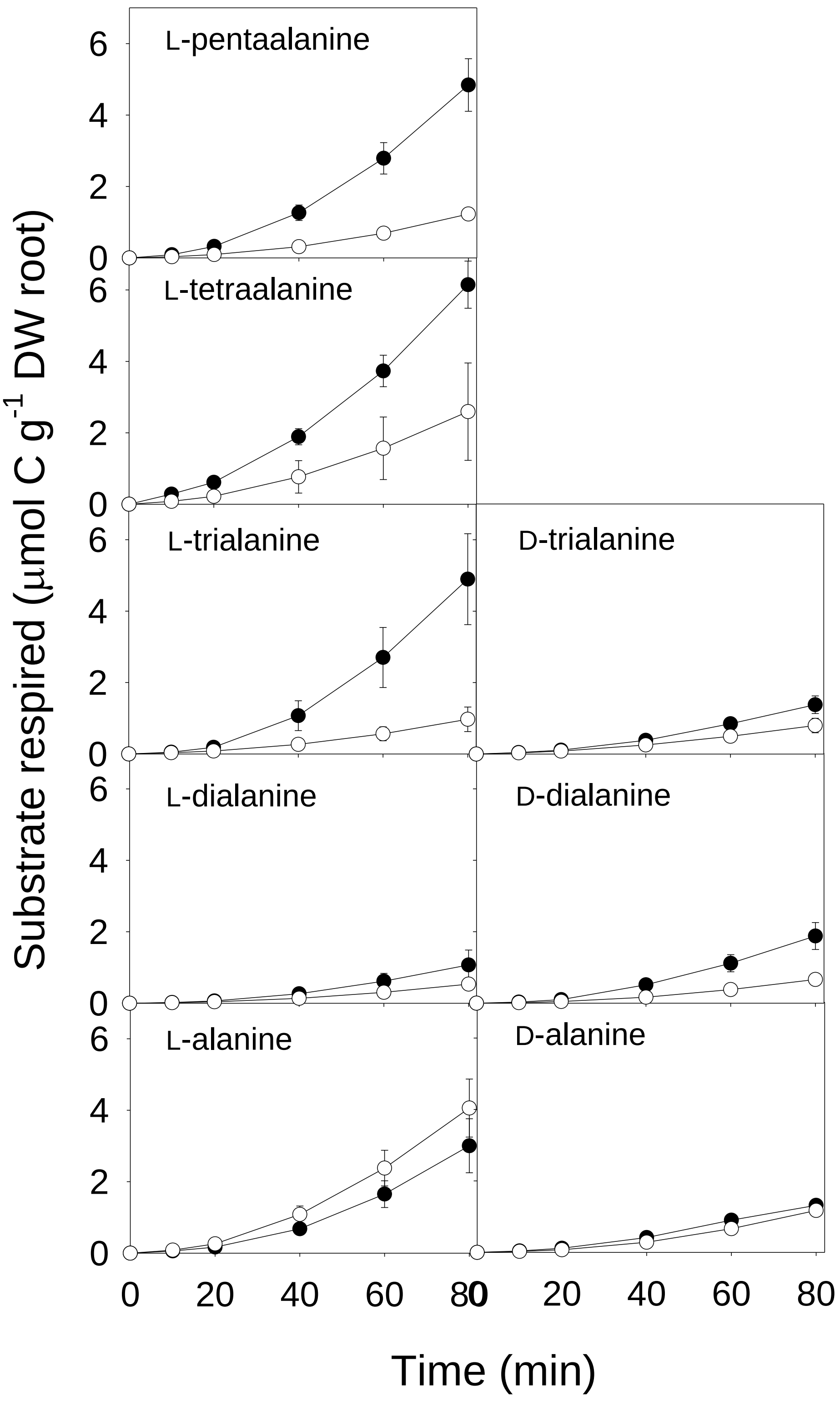

Figure S7 Loss in respiration of C supplied to roots of *D. antarctica* as D- and L-alanine and their short peptides. Closed and open circles are plants colonised with DSE and unicolulated controls, respectively. Data are mean ± SEM; *n*=3.

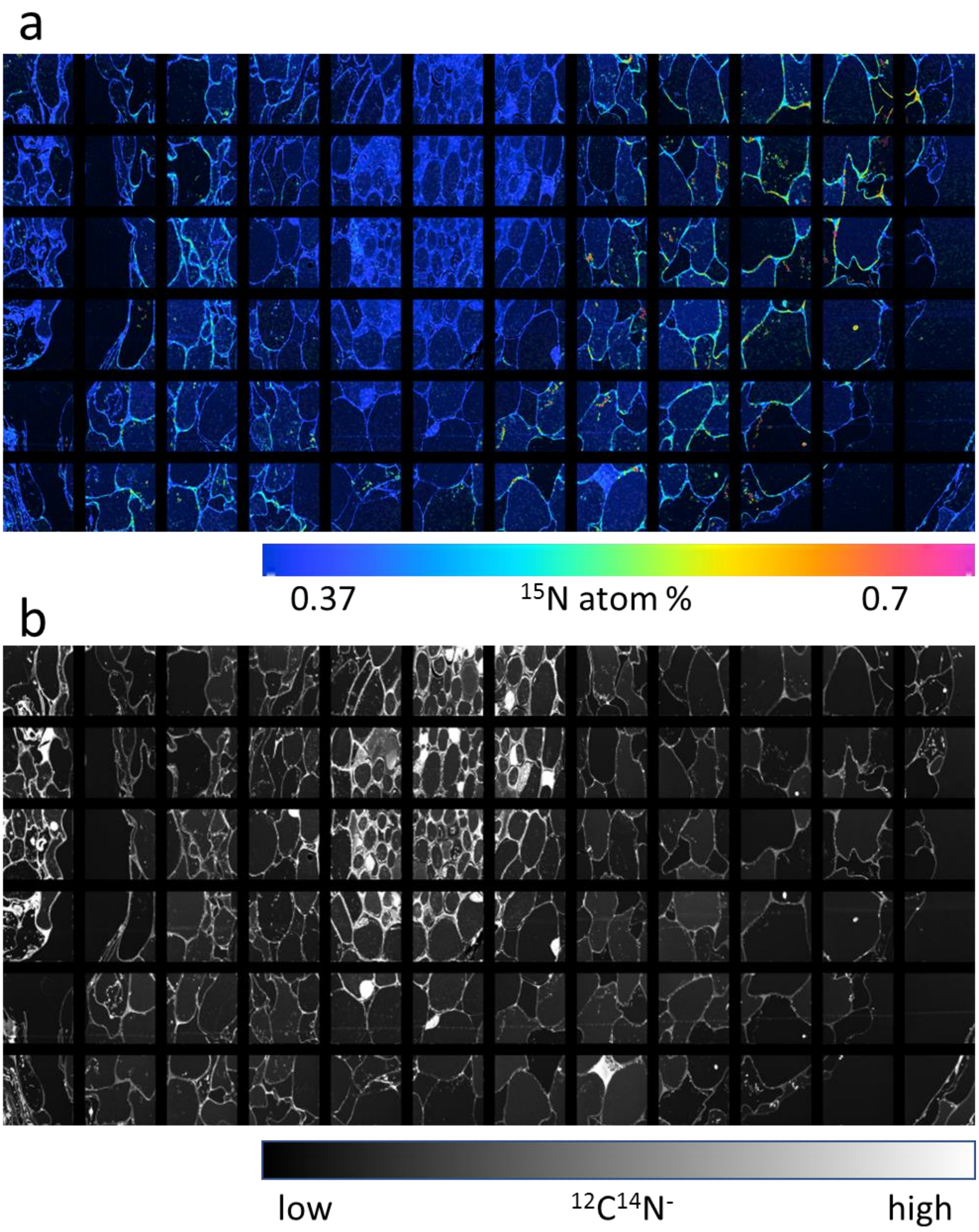

Figure S8 ^{15}N enrichment image of entire root cross-section of *D. antarctica* inoculated with DSE and incubated for 5 min in ^{15}N L-trialanine. Similar montaged images were generated for all four treatments (+/-DSE, D or L ^{15}N-trialinine) and an unlabelled control. For the example displayed here (A), ^{15}N enrichment is highest in the intercellular spaces of the cortical zone and also in portions of microsclerotia. B) $^{12}C^{14}N^{-}$ ion (proxy for ^{14}N) intensity image of the same area is included as a reference to sample ultrastructure.

Table S1 Michaelis-Menten constants for uptake of various forms of N by roots of *D. antarctica* without or with DSE colonisation

	Km ($\mu mol\ l^{-1}$)		Vmax ($\mu mol\ g^{-1}$ DW root h^{-1})		Difference between DSE colonised and control plants
	-DSE	+DSE	-DSE	+DSE	
NO_3^-	3488	3308	42.0	36.8	P=0.90
NH_4^+	5743	5191	35.9	42.2	P=0.007
L-alanine	323.1	840.6	12.5	42.7	P=0.07
D-alanine	657.1	782.3	15.2	20.6	P=0.04
L-dialanine	222.2	410.9	11.1	45.3	P=0.02
D-dialanine	1261	663.4	27.0	23.2	P=0.08
